# Are there associations between religious affiliation and drive for muscularity? A cross-sectional survey of young Muslim women, Christian women and atheist women from Germany

**DOI:** 10.1186/s12905-020-01138-8

**Published:** 2020-12-09

**Authors:** Leonie Wilhelm, Andrea S. Hartmann, Julia C. Becker, Manuel Waldorf, Silja Vocks

**Affiliations:** 1 Department of Clinical Psychology and Psychotherapy, Universität Osnabrück, Osnabrück, Germany; 2 Department of Social Psychology, Universität Osnabrück, Osnabrück, Germany

**Keywords:** Veiling, Sports, Body image, Leanness, Muscularity

## Abstract

**Background:**

Over the last 20 years, society’s perception of the ideal female body size in Western cultures has changed from thin to athletic, and many women practice sports to achieve well-toned bodies. However, to date, no study has investigated whether Muslim women who live in a Western country and veil their bodies strive for lean or muscular bodies too. The current cross-sectional survey therefore addressed this question.

**Methods:**

Veiled Muslim women (*n* = 70), unveiled Muslim women (*n* = 50), Christian women (*n* = 79), and atheist women (*n* = 68) living in Germany answered several questionnaires assessing engagement in sports, body appreciation, and drive for leanness and muscularity. Univariate and multivariate analyses were conducted to compare the four groups.

**Results:**

The results of univariate and multivariate analyses showed that Muslim women engaged less in sports and veiled Muslim women reported higher body appreciation than did Christian and atheist women. Although the groups did not differ significantly in drive for muscularity, Muslim women showed lower levels of drive for leanness than did Christian and atheist women.

**Conclusion:**

Given that Muslim women engaged less in sports and strived less for a lean body compared to Christian and atheist women, a well-toned body might be less important for them. Nevertheless, as being active is beneficial for general health, barriers that prevent Muslim women from engaging in sports should be diminished.

## Background

According to the Olympic Movement, the practice of sport is a human right, and nobody should be discriminated against due to their gender or religion [1]. However, worldwide, fewer women than men do sports, and depending on country, culture, and sporting activity, women are prevented or even forbidden from engaging in sports [2]. Underlying reasons for this gender difference in sports engagement are of a cultural (e.g., sport and its representation in the media is typically male-dominated; [3]), religious (e.g., Islamic body-covering and gender segregation impede participation in sports; [4–6]), evolutionary (e.g., men are more physically competitive; [7]), social (e.g., influence of family or socioeconomic status; [5, 6, 8]), or practical nature (e.g., suitable facilities or equipment; [4, 6]).

In general, being physically active enhances women’s self-esteem and promotes a positive body image [9]. Body image is defined as a person’s mental representation of his/her own body and the feelings regarding this mental representation [10]. Accordingly, a person’s body image might be favorable and positive, resulting in high body appreciation, or unfavorable and negative, leading to a negative attitude towards one’s own body [11]. As a negative body image is a risk factor for developing an eating disorder [12], it is important to identify factors which positively influence body image, such as engaging in sports [9].

Sporting activity is associated with higher levels of body esteem and self-esteem, and lower levels of depression [13]. In turn, higher levels of self-esteem correlate with higher levels of body appreciation [11, 14]. Thus, women who are more active also think more positively about their bodies and feel more self-confident. For instance, in contrast to non-athletes, athletes reported lower levels of body dissatisfaction [15]. Likewise, in an experimental study with a cross-over design, female participants were randomized to two groups, beginning with either endurance training or with reading a newspaper, and reported greater body satisfaction after endurance training [16]. However, it is important to note that depending on the underlying motivation (e.g., to lose weight, to enjoy), physical exercise can enhance both a positive and a negative body image [17, 18].

For instance, many women exercise not only in order to feel good, but also to achieve a lean and muscular body, as over the last 20 years [19], the Western beauty ideal for women has changed from being very thin to having an athletic body (see 13 for a review). According to Smolak and Murnen [20], the ideal lean body has well-toned muscles and little body fat. Trends such as “fitspiration” are ubiquitous in social media and motivate women to exercise in order to obtain a lean body [21]. In contrast, a muscular body is heavier as it consists of a higher proportion of muscle mass [22]. According to women’s magazines focusing on fashion and fitness, thinness and leanness are two desirable features for a female body [23]. With regard to thinness, processes such as Westernization and modernization as well as the influence of the mass media have led to a narrowing of differences in the body size ideals between Western and non-Western countries, resulting in a global thin ideal [24, 25]. In this context, it is important to keep in mind that in some countries, such as Mauritania, women are partly expected to be heavier [26]. Nevertheless, over the last decades, the ideal for the female body has changed in many countries; nowadays, women from Arab countries such as Bahrain, Egypt, Jordan, Oman and Syria show a similar preference for a thin body size to women from the United States [27]. Thus, most women want to have a thin body, irrespective of their cultural background. Research on female students from Kuwait showed that the consumption of mass media is negatively linked to women’s body image, especially in obese women [28]. As recent research found associations between the internalization of the thin ideal and the internalization of the athletic ideal [23], it might be possible that a global leanness ideal will also develop in the coming years. However, most of the respective studies focused on white females, with the majority being Christian (e.g., [3, 21]), and no empirical study to date has investigated drive for leanness and drive for muscularity in Muslim women. Therefore, it is of interest to examine whether Muslim women also strive for a lean and muscular body. Like in other European countries, e.g., Norway [29], the majority of Muslims living in Germany are Sunni (74%) and have a Turkish migration background (63%); only persons with an Iranian migration background are predominantly Shia Muslims [30]. With regard to the research question of the present study (i.e., belief contents with a potential influence on body image, religious practices that potentially interfere with exercise behavior), Sunni and Shia Muslims in Germany do not seem to differ in a meaningful way [30]. Therefore, and with regard to the sample size, no distinction was made between Sunni and Shia Muslims in the current study.

Although Islam acknowledges the importance of exercise in order to stay healthy [31, 32] and the Prophet Muhammad recommended sports like swimming and running [33], researchers from the Netherlands found that compared to other women from ethnic minority groups, Muslim women engaged less in sports [34]. Recent studies indicated that Muslim women are often prevented from doing sports [1, 6]. For example, some Muslim women do not want to engage in sporting activities because their Muslim peers, relatives, or neighbors disapprove of Muslim women engaging in sport or even exercising in mixed teams of men and women [32, 35, 36]. In many traditional Muslim households, women are expected to focus on their religion and to marry and have children at a very young age [37]. Moreover, in contrast to Muslim men, Muslim women’s prestige and family honor is often related to chastity before marriage [38]. Norwegian Muslim women reported that they do not engage in sports because it is not seen as respectable for Muslim women to do sports, especially competitive sports [29]. Moreover, some Muslim women are not allowed to engage in sporting activities [5, 6, 35] or cannot find suitable facilities to do sports [5]. In the case of Muslim women who veil or cover their bodies (e.g., with a hijab or a chador), difficulties with their veiling is often stated as a reason for not engaging in sporting activities [1, 5, 39]. While the hijab covers only the neck and the hair, the chador is a long, loose covering from head to feet; however, the face is not covered [6, 40]. Especially in the past, some professional Muslim athletes have suffered from regulations that forbid the wearing of modest clothing, such as the hijab, during competitions [5], although women like the sprinter Ruqaya Al-Ghasara from Bahrain have demonstrated that it is possible to take part in the Olympic Games while wearing the Islamic body covering [1].

All of these reasons have resulted in a low percentage of Muslim women—and especially veiled Muslim women—doing sports [5, 41]. However, despite various barriers, Muslim women do find individual ways to exercise (e.g., dancing, doing sit-ups at home or playing soccer with siblings; [42, 43]), and it is important to note that many Muslim parents support their daughters’ sporting activities [4, 32, 42, 44]. Generally speaking, Muslim women who are more active report greater levels of psychological well-being and lower levels of depression than do Muslim women who are less active [45], and women from Tunisia reported that exercising decreases their stress levels [46].

Most of the previous research on Muslim women’s sporting behavior is of a qualitative nature and included only a small number of participants [32, 42, 43] For instance, in semi-structured interviews, 11 young Muslim women from Australia described the physical activities they do on a day-to-day basis, e.g., playing soccer with siblings [42]. A similar finding emerged from another qualitative study that compared young Muslim women’s experiences regarding physical education between Greek Turkish Muslim women and British Asian Muslim women [4]. When asked about physical activity in their leisure time, many of the interviewed participants reported playing games with friends [4]. However, there are a few quantitative studies on Muslim women’s physical activity; for instance, the weekly level of physical activity in Muslim mothers of young children living in the United States was assessed via a two-item tool [47, 48]. It is important to note that the majority of studies did not differentiate between veiled and unveiled Muslim women, even though research has found that engaging in sports is more difficult for women who wear the hijab compared to women who do not [5, 47]. Therefore, quantitative studies comprising a greater number of participants are warranted in order to examine Muslim women’s actual engagement in sports, that is, how much time they spend doing sports in an average week. Furthermore, as very little research has examined the relationship between sports and religion [49], it is of interest to compare the engagement in sports between Muslim women and women of other religious affiliations or non-religious women.

The present cross-sectional survey therefore compared the engagement in sports of veiled and unveiled Muslim women, Christian women, and atheist women living in Germany. Additionally, the four groups were compared regarding their levels of body appreciation, self-esteem, and drive for thinness, leanness, and muscularity. Moreover, relationships between engagement in sports, positive body image, and self-esteem were investigated and compared between the four groups. In line with studies on barriers impeding Muslim women from engaging in sporting activities (e.g., [1]), it was hypothesized that Muslim women would engage less in sports compared to Christian and atheist women (hypothesis 1). Given previous findings of a positive relationship between sports and body esteem as well as self-esteem [13], it was expected that a higher engagement in sports to be associated with higher levels of body appreciation and self-esteem (hypothesis 2). As research on body image revealed that women with a more positive body image report higher levels of self-esteem than those with a more negative body image [11, 14], a positive correlation between body appreciation and self-esteem in each of the four groups was expected (hypothesis 3). Finally, given that Muslim women rarely engage in sports [34], it was hypothesized that Muslim women strive less for a lean and muscular body compared to Christian and atheist women (hypothesis 4).

## Methods

### Participants

The present study exclusively included females aged 16–35 years, as young women’s body image has been found to differ from that of middle-aged women [50]. In total, 400 questionnaires were distributed, of which 319 were returned (79.8%). On the first page of the questionnaire, participants were asked to agree to the conditions of data assessment. Finally, participants had to self-define as atheist, Christian or Muslim. Based on these inclusion criteria, *n* = 1 (14-year-old woman), *n* = 43 women (aged over 35 years), and *n* = 1 (woman who did not state her age) were excluded. The further exclusion of *n* = 5 (women did not agree to the conditions of data assessment) and *n* = 2 women (did not self-define as atheist, Christian or Muslim) resulted in *n* = 267 women for the final data analysis. Depending on religious affiliation and in the case of Muslim women depending on Islamic body covering (see the following Hijab Index; [40]), participants were divided into four groups, resulting in *n* = 70 veiled Muslim women, *n* = 50 unveiled Muslim women, *n* = 79 Christian women and *n* = 68 atheist women.

### Measures

#### Hijab Index

The ‘Hijab Index’ [40], that we got directly from the authors, assesses the frequency (“How frequently do you wear an Islamic headscarf (e.g., hijab, chador, burqa, etc.)?”) and conservativeness of Islamic body covering. It was used to determine whether and how Muslim women cover their bodies in public in order to distinguish between veiled and unveiled Muslim women. The frequency dimension was rated on a 5-point scale from 1 (*never*) to 5 (*always*) and the conservativeness dimension was rated on a 7-point scale from 0 *(no covering*) to 6 (*long covering from head to feet, and face covered completely*). Besides written descriptions, six drawings displaying various styles of hijab were given as response options. A Hijab Index score was created by multiplying hijab frequency and hijab conservativeness, with a possible range from 0 to 30. Additionally, Muslim women reported their individual reasons for wearing the hijab and/or selected one or more of the provided options based on previous literature [40]. Furthermore, participants stated the age at which they began to cover their bodies and reported for how many hours they usually cover their bodies on an average day. In the current study, Muslim women who covered their bodies for more than one hour per day and who had Hijab Index scores greater than 2 were categorized as veiled Muslim women. In contrast, Muslim women who covered their bodies for only one hour or less per day (e.g., only when praying) and thus had Hijab Index scores of 2 or less were defined as unveiled Muslim women, as veiling during prayer does not affect their engagement in sports.

#### Eating Disorder Examination-Questionnaire

The license-free Eating Disorder Examination-Questionnaire ([51]; German-language version: [52]) assesses the intensity and frequency of the main features of eating disorders during the past 28 days. The questionnaire comprises the four subscales *Restraint* (5 items), *Eating concern* (5 items), *Shape concern* (8 items), and *Weight concern* (5 items), and its items were rated on a 7-point Likert scale ranging from 0 (*no days/not at all*) to 6 (*every day/markedly*). Given Cronbach’s alphas ranging from α = 0.85 to α = 0.97 [53], the questionnaire can be considered as internally consistent. Regarding the present study, Cronbach’s alpha ranged from α = 0.80 to α = 0.92 for the four subscales.

#### Engagement in sports

Following previous research [54, 55], we assessed the frequency as well as the duration of participants’ physical exercise using two single items. Participants first indicated the number of times they engaged in physical exercise in an average week (< 1 times, 1–3 times, 4–6 times, > 6 times) and then indicated the average duration of a single exercise session (< 30 min, 31–60 min, 61–120 min, > 120 min). The respective items developed for this study are provided as additional file (see Additional file 1).

#### Body Appreciation Scale

The self-report Body Appreciation Scale-2 ([56]; German-language version: [57]) assesses positive facets of body image (example item: “I feel good about my body.”). The 10 items were rated on a 5-point Likert scale ranging from 1 (*never*) to 5 (*always*). Excellent consistencies for the scale were reported by the authors (α = 0.94) and in the present study (α = 0.93). The authors gave permission to use the German-language version of the Body Appreciation Scale-2.

#### Rosenberg Self-Esteem Scale

The Rosenberg Self-Esteem Scale ([58]; German-language license-free version: [59]) was included to determine participants’ global self-esteem. This self-report measure consists of 10 items (example item: “I feel that I'm a person of worth.”) rated on a 4-point Likert scale ranging from 0 (*strongly disagree*) to 3 (*strongly agree*). Good internal consistencies were reported by the authors (α = 0.81 to α = 0.87) and in the current study (α = 0.89).

#### Drive for Thinness

The subscale *Drive for Thinness* from the Eating Disorder Inventory 2 ([60]; German-language license-free version: [61]) was used to assess participants’ urge to be thin. The 7 items of the scale (example item: “I am preoccupied with the desire to be thinner.”) were rated on a 6-point Likert scale ranging from 1 (*never*) to 6 (*always*). Good internal consistencies for the scale were reported by the authors (α = 0.81 to α = 0.91) and in the present study (α = 0.92).

#### Drive for Leanness

The Drive for Leanness scale [20] consisting of 6 items was used to determine participants’ desire for a well-toned body. The items (example item: “I think the best bodies are well-toned.”) were rated on a 6-point Likert scale ranging from 1 (*never*) to 6 (*always*). Good internal consistencies were reported by the authors (α = 0.71) and in the present study (α = 0.84). The 6 items were professionally translated into German for a previous study of our lab (see [62]).

#### Drive for Muscularity Scale

The Drive for Muscularity Scale ([63]; German-language version: [64]), that we got directly from the authors, assesses respondents’ urge to be muscular and to gain weight. It consists of 15 items (example item: “I wish that I were more muscular.”) rated on a 6-point Likert scale ranging from 1 (*always*) to 6 (*never*). Cronbach’s alpha in the current study (α = 0.83) is in line with the reported internal consistency for women (α = 0.82; [22]).

### Procedure

The data were collected between January 2017 and February 2019. Co-workers and student assistants tested the questionnaires in advance with respect to comprehensibility. Participants were either recruited through different mailing lists (e.g., universities, Muslim associations), advertisements on Facebook, or personal conversations. No cover story was used; potential participants were told that the study was about engagement in sports, body image as well as drive for leanness and for muscularity. Interested participants then contacted the study director and the questionnaires were either completed in the lab of the university or were sent to participants by post. Psychology students who completed the questionnaires in the lab were given course credits as reimbursement; the postal questionnaires included stamped addressed envelopes and a code that participants could send to the study director via email in order to receive a 5€ voucher from an online retailer. On the first two pages of the questionnaire, participants were informed about the study, asked to provide their consent to participate, and were told that the questions assess demographic information, eating behavior, and thoughts and feelings about their shape and their body. To begin with, participants stated their age, height, weight, educational background, family status, number of children, country of birth, and religious affiliation. Women who identified themselves as Muslim also completed the Hijab Index. Next, all participants completed the various questionnaires. The study was approved by the local ethics committee.

### Statistical analyses

First, demographic characteristics such as age, body mass index, country of birth, relationship status, number of children, educational background, and eating behavior were compared between the four groups by conducting univariate analyses, χ^2^-tests, and a Kruskal–Wallis-H-test. In the case of eating behavior, a multivariate analysis of variance (MANOVA) was conducted. Second, only in the case of veiled Muslim women, mean analyses were calculated to determine the age at which women started to veil and and the average number of veiling hours per day. Furthermore, frequency analyses were conducted to ascertain the women’s reasons for their veiling. In order to test whether Muslim women engage less in sports compared to Christian and atheist women (hypothesis 1), a χ^2^-test was used to examine whether the number of women who do not engage in any sports differed significantly between the four groups. Then, Kruskal–Wallis-H-tests were conducted to compare the weekly amount of physical exercise and the average length of a single exercise session between the four groups. As positive links between engagement in sports, body appreciation and self-esteem were expected (hypotheses 2 and 3), Pearson correlation coefficients and Spearman’s rank correlation coefficients were calculated to examine the relationships between engagement in sports, body appreciation, and self-esteem. Additionally, two univariate analyses of variance (ANOVAs) were conducted in order to compare the four groups regarding levels of body appreciation and self-esteem. Finally, given the hypothesis that Muslim women strive less for a lean and muscular body compared to Christian and atheist women (hypothesis 4), a MANOVA was conducted to compare the groups’ scores on the Drive for Leanness, Drive for Thinness, and Drive for Muscularity scales. The Statistical Package for the Social Sciences SPSS 24 (IBM; Armonk, United States) was used for all statistical analyses. Missing data was not imputed; the respective incomplete score was not included in the analysis. In general, *p* < .05 (two-tailed), which was corrected for multiple comparisons, indicated statistical significance. When results reached significance, Bonferroni-adjusted post hoc comparisons, i.e., Hochberg GT2, Games-Howell, Mann–Whitney-U-test, were conducted. As effect sizes, η_p_^2^ or Cohen’s *d* were used, and according to conventional classifications, η^2^ = 0.01 and *d* = 0.2 indicate small, η^2^ = 0.06 and *d* = 0.5 indicate medium, and η^2^ = 0.12 and *d* = 0.8 indicate large effects, respectively [65, 66].

## Results

### Group characteristics

The four groups differed in terms of age, country of birth, relationship status, number of children, and educational status, but not regarding body mass index. Furthermore, the groups did not differ significantly regarding their levels of restraint, eating concern, shape concern, and weight concern, Wilks’ λ = 0.940, *F*(12, 685.541) = 1.361, *p* = .180, η_p_^2^ = 0.02 (see Table 1). Christian women were slightly older compared to veiled Muslim women, *p* = .001, *d* = 0.65, and unveiled Muslim women, *p* = .028, *d* = 0.32, and atheist women were older than veiled Muslim women, *p* = .003, *d* = 0.62. Compared to veiled Muslim women, *p* < .001, *d* = 0.67, and unveiled Muslim women, *p* = .001, *d* = 0.64, a higher percentage of Christian women stated Germany as their country of birth. Atheist women were more often born in Germany than were veiled Muslim women, *p* = .001, *d* = 0.59. Given that the time of acculturation to Western standards might have led to differences in body image and engaging sports, all of the statistical analyses were repeated between women who were and were not born in Germany as a proxy for time of acculturation. None of the comparisons reached significance, with the exception of engaging in sports: Compared to women who were not born in Germany, women who were born in Germany were more likely to engage in sports, χ^2^ = 76.19, *p* < .000, *d* = 1.29. H owever, it is important to note that the group of women who were not born in Germany was much smaller than the group born in Germany. Compared to veiled Muslim women, *p* < .001, *d* = 0.80, and unveiled Muslim women, *p* = .009, *d* = 0.47, a higher percentage of Christian women reported currently being in a romantic relationship. In addition, more atheist women than veiled Muslim women, *p* < .001, *d* = 0.72, and unveiled Muslim women, *p* = .033, *d* = 0.40, reported being in a relationship. With regard to number of children, Christian women had more children than did atheist women, *p* = .015, *d* = 0.58. Atheist women had a higher educational status than did veiled Muslim women, *p* < .001, η^2^ = 0.10, unveiled Muslim women, *p* = .006, η^2^ = 0.06, and Christian women, *p* < .001, η^2^ = 0.09.

**Table 1:** Groups’ characteristics (N = 267)

Variable	Veiled Muslim women (*n* = 70)		Unveiled Muslim women (*n* = 50)		Christian women (*n* = 79)		Atheist women (*n* = 68)		Group comparison			
	*M*	*SD*	*M*	*SD*	*M*	*SD*	*M*	*SD*	*F*	*df*	*p*	η_p_^2^
Mean age (in years)	22.01^c,d^	3.58	22.30^c^	5.42	25.13^a,b^	5.71	24.44^a^	4.30	7.08	3	< .001**	0.08
Mean body mass index^e^	23.35	4.03	23.43	4.55	23.05	4.91	22.43	3.55	0.71	3	.545	0.01
Number of participants												
Born in Germany^f^	55^c,d^ (78.6%)		40^c^ (80.0%)		73^a,b^ (92.4%)		63^a^ (92.7%)		χ^2^ = 20.63		< .001**	
With a current relationship	16^c,d^ (22.9%)		18^c,d^ (36.0%)		47^a,b^ (59.5%)		38^a,b^ (55.9%)		χ^2^ = 25.49		< .001**	
Number of children^g^	0.13	0.51	0.16	0.58	0.34^d^	0.82	0.04^c^	0.27	3.36	3	.019*	0.04
Highest qualification: Number of participants with									*H*(3) = 17.20		.001*	η^2^ = 0.06
Intermediate school degree	9 (12.9%)		10 (20.0%)		9 (11.4%)		0 (0%)					
High school degree	46 (65.7%)		24 (48.0%)		53 (67.1%)		38 (55.9%)					
University degree	12 (17.1%)		14 (28.0%)		17 (21.5%)		28 (41.2%)					
Eating Disorder Examination-Questionnaire^h^												
Restraint	0.96	1.00	1.32	1.42	1.45	1.36	1.39	1.34	2.14	3	.096	0.03
Eating concern	0.52	0.82	0.92	1.16	0.72	0.84	0.76	1.01	1.81	3	.146	0.02
Shape concern	1.68	1.41	2.29	1.70	2.20	1.52	2.15	1.43	2.15	3	.095	0.02
Weight concern	1.42	1.19	1.96	1.54	2.04	1.43	1.85	1.41	2.78	3	.042	0.03

**p < .001; *p < .05

^a^Differ significantly from veiled Muslim women (*p* < .05)

^b^Differ significantly from unveiled Muslim women (*p* < .05)

^c^Differ significantly from Christian women (*p* < .05)

^d^Differ significantly from atheist women (*p* < .05)

^e^One unveiled Muslim woman did not report her weight

^f^One unveiled Muslim woman, five Christian women, and two atheist women did not report their country of birth

^g^One veiled Muslim woman did not report her number of children

^h^One unveiled Muslim woman was not included in the analysis because of missing data

### Hijab Index

Veiled Muslim women began to cover their bodies at the age of *M* = 15.14 (*SD* = 3.71). On average, they veil their bodies for *M* = 7.78 (*SD* = 3.17) hours per day. All of the *n* = 70 veiled Muslim women (100%) stated “religious reasons” as one reason for their veiling, 48.6% stated “to protect my body against the view of others”, and 30.0% stated “to feel comfortable in mixed-sex settings”. Furthermore, 24.3% selected “to show others my Muslim identity” and 7.1% stated “to protect against Western ideas or lifestyle” and “cultural reasons”, respectively. Finally, one woman selected “a wish of my family”.

### Engagement in sports

The χ^2^-test investigating whether the number of women who do no sports differs between the groups was significant, χ^2^ = 24.27, *p* < .001 (see Table 2). Subsequent χ^2^-tests revealed that compared to Christian women, *p* < .001, *d* = 0.64, and atheist women, *p* < .001, *d* = 0.76, a higher percentage of veiled Muslim women did no sports. Additionally, a higher percentage of unveiled Muslim women did no sports compared to Christian women, *p* = .019, *d* = 0.42, and atheist women, *p* = .004, *d* = 0.56.

**Table 2:** Participants’ reports on engagement in sports by groups (N = 267)

	Veiled Muslim women (*n* = 70)	Unveiled Muslim women (*n* = 50)	Christian women (*n* = 79)	Atheist women (*n* = 68)
No sports	32 (45.7%)^c,d^	18 (36.0%)^c,d^	14 (17.7%)^a,b^	9 (13.2%)^a,b^
Amount of exercise per week				
< 1 times	43 (61.4%)	23 (46.0%)	20 (25.3%)	21 (30.9%)
1–3 times	22 (31.4%)	24 (48.0%)	47 (59.5%)	38 (55.9%)
4–6 times	3 (4.3%)	2 (4.0%)	12 (15.2%)	8 (11.8%)
> 6 times	2 (2.9%)	1 (2.0%)	0 (0%)	1 (1.5%)
Average duration of exercise				
< 30 min	40 (57.1%)	19 (38.0%)	20 (25.3%)	10 (14.7%)
31–60 min	22 (31.4%)	20 (40.0%)	29 (36.7%)	40 (58.8%)
61–120 min	8 (11.4%)	11 (22.0%)	28 (35.4%)	17 (25.0%)
> 120 min	0 (0%)	0 (0%)	2 (2.5%)	1 (1.5%)

^a^Differ significantly from veiled Muslim women (*p* < .05)

^b^Differ significantly from unveiled Muslim women (*p* < .05)

^c^Differ significantly from Christian women (*p* < .05)

^d^Differ significantly from atheist women (*p* < .05)

Regarding the weekly amount of physical exercise, the Kruskal–Wallis-H-test was significant, Kruskal–Wallis-test *H*(3) = 21.80, *p* < .001, η^2^ = 0.07. Subsequent pairwise Mann–Whitney-U-tests showed that veiled Muslim women reported a smaller amount of weekly exercise compared to Christian women, *p* < .001, η^2^ = 0.12, and atheist women, *p* = .006, η^2^ = 0.08. With regard to the duration of a single exercise session, the Kruskal–Wallis-H-test was significant, Kruskal–Wallis-test *H*(3) = 29.12, *p* < .001, η^2^ = 0.10. Subsequent pairwise Mann–Whitney-U-tests revealed that veiled Muslim women had a shorter average duration of exercise compared to Christian women, *p* < .001, η^2^ = 0.13, and atheist women, *p* < .001, η^2^ = 0.17.

As can be seen in Table 3, the correlations between engagement in sports and body appreciation as well as between engagement in sports and self-esteem were significant only in the case of veiled Muslim women and Christian women.

**Table 3:** Correlations between engagement in sports (Eng. in sports), body appreciation, and self-esteem by groups (N = 267)

Correlation between	Veiled Muslim women (*n* = 70)	Unveiled Muslim women (*n* = 50)	Christian women (*n* = 79)	Atheist women (*n* = 68)
Eng. in sports	*r*_*s*_ = .28*	*r*_*s*_ = .11	*r*_*s*_ = .28*	*r*_*s*_ = − .06
Body appreciation	(*n* = 66)	(*n* = 49)	(*n* = 79)	(*n* = 66)
Eng. in sports	*r*_*s*_ = .29*	*r*_*s*_ = .13	*r*_*s*_ = .23*	*r*_*s*_ = .02
Self-esteem	(*n* = 68)	(*n* = 47)	(*n* = 76)	(*n* = 67)
Body appreciation	*r* = .69**	*r* = .48*	*r* = .71**	*r* = .74**
Self-esteem	(*n* = 65)	(*n* = 47)	(*n* = 76)	(*n* = 66)

*p < .05; **p < .001

### Body Appreciation Scale-2

The ANOVA was significant, *F*(3,256) = 6.362, *p* < .001, η_p_^2^ = 0.07, with subsequent post hoc tests showing that veiled Muslim women reported higher body appreciation than did Christian women, *p* < .001, *d* = 0.68, and atheist women, *p* = .004, *d* = 0.61 (see Table 4).

**Table 4:** Participants’ mean scores on body appreciation, self-esteem, and drive for leanness, thinness, and muscularity (N = 267)

Variable	Veiled Muslim women (*n* = 70)		Unveiled Muslim women (*n* = 50)		Christian women (*n* = 79)		Atheist women (*n* = 68)		Analysis of variance			
	*M*	*SD*	*M*	*SD*	*M*	*SD*	*M*	*SD*	*F*	*df*	*p*	η_p_^2^
Body appreciation^e^	3.83^c,d^	0.73	3.55	0.77	3.33^a^	0.75	3.38^a^	0.71	6.36	3	< .001**	0.07
Self-esteem^f^	23.43	5.54	22.38	5.98	22.82	5.72	21.21	6.36	1.71	3	.165	0.20
Drive for leanness^g^	16.02^c,d^	5.31	16.75^c,d^	5.31	19.77^a,b^	5.50	19.65^a,b^	5.71	8.23	3	< .001**	0.09
Drive for thinness^g^	2.56	1.22	2.86	1.41	3.04	1.34	2.85	1.24	1.66	3	.176	0.02
Drive for muscularity^g^	1.85	0.58	1.89	0.60	2.01	0.65	1.99	0.62	1.06	3	.368	0.01

**p < .001; *p < .05

^a^Differ significantly from veiled Muslim women (*p* < .05)

^b^Differ significantly from unveiled Muslim women (*p* < .05)

^c^Differ significantly from Christian women (*p* < .05)

^d^Differ significantly from atheist women (*p* < .05)

^e^Four veiled Muslim women, one unveiled Muslim woman, and two atheist women were not included in the analysis because of missing data

^f^Two veiled Muslim women, three unveiled Muslim women, three Christian women, and one atheist woman were not included in the analysis because of missing data

^g^Five veiled Muslim women, three unveiled Muslim women, and two Christian women were not included in the analysis because of missing data

### Rosenberg Self-Esteem Scale

As shown in Table 4, the ANOVA was not significant. Thus, the four groups did not differ significantly regarding their levels of self-esteem. However, correlations between self-esteem and body appreciation were significant in each of the four groups (see Table 3).

### Drive for Thinness, Drive for Leanness, and Drive for Muscularity

The MANOVA was significant, Wilks’ λ = 0.90, *F*(9, 611.019) = 3.09, *p* = .001, η_p_^2^ = 0.04. The subsequent ANOVA regarding *Drive for Thinness* was not significant, *F*(3,257) = 1.66, *p* = .176, η_p_^2^ = 0.02. Thus, drive for thinness did not differ significantly between the four groups. However, the subsequent ANOVA regarding *Drive for Leanness* was significant, *F*(3,257) = 8.23, *p* < .001, η_p_^2^ = 0.09. Subsequent post hoc tests revealed that Christian women showed a greater drive for leanness compared to veiled Muslim women, *p* < .001, *d* = 0.69, and unveiled Muslim women, *p* = .019, *d* = 0.56. In addition, atheist women reported a greater drive for leanness than did veiled Muslim women, *p* = .001, *d* = 0.66, and unveiled Muslim women, *p* = .033, *d* = 0.52. As the subsequent ANOVA for *Drive for Muscularity* was not significant, *F*(3,257) = 1.06, *p* = .368, η_p_^2^ = 0.01, drive for muscularity did not differ significantly between the four groups (see Table 4).

## Discussion

The present study contributed to the limited quantitative research on how much Muslim women engage in sports [31, 43] by comparing veiled and unveiled Muslim women, Christian women, and atheist women regarding their engagement in sports. Moreover, the study was the first to investigate drive for leanness and drive for muscularity in Muslim women.

In sum, it was found that a higher percentage of veiled and unveiled Muslim women do not engage in sporting activities compared to Christian women and atheist women. In addition, veiled Muslim women reported a lower weekly amount of physical exercise than did Christian women and atheist women. Therefore, the first hypothesis, stating that Muslim women would show less engagement in sports than Christian women and atheist women, was confirmed. In contrast, the second hypothesis, that a higher engagement in sports would be linked to higher levels of body appreciation and self-esteem, was only confirmed for veiled Muslim women and Christian women, as mixed results emerged regarding the correlations between engagement in sports, body appreciation, and self-esteem. In the case of veiled Muslim women and Christian women, engagement in sports correlated significantly with levels of body appreciation and self-esteem. In contrast, for unveiled Muslim women and atheist women, no significant correlations emerged between engagement in sports and body appreciation or self-esteem. However, the correlation between body appreciation and self-esteem was significant across the four groups, thus confirming the third hypothesis. With regard to the fourth hypothesis, which stated that Muslim women would show a lower urge for a lean and muscular body than Christian women and atheist women, the findings were mixed. While the four groups did not differ significantly regarding drive for muscularity, veiled and unveiled Muslim women reported lower levels of drive for leanness than did Christian women and atheist women. Therefore, hypothesis four was only partially confirmed. Moreover, veiled Muslim women reported higher body appreciation than did Christian women and atheist women, but no significant group differences emerged regarding self-esteem and drive for thinness.

In terms of the very large number of Muslim women, and especially veiled Muslim women, who do not engage in any sports, one might assume that the veiling might impede engagement in sports [5, 47]. The finding of the present study is in line with previous research on college students stating that Muslim women from Jordan, Pakistan, and Oman engaged less in sports than did Christian and atheist women from the US [18, 36]. Given the harmful effects of being inactive on health [67, 68], it is important to investigate possible reasons for the low sporting participation rates of Muslim women in order to provide a basis on which to develop selective prevention programs.

Interestingly, although Muslim women do engage less in sporting activities compared to Christian and atheist women, veiled Muslim women reported higher body appreciation than did Christian women and atheist women. In view of the positive associations between wearing the hijab and a positive body image found in recent research [69, 70], it is possible that veiling might protect Muslim women’s body image against negative psychological outcomes. However, as veiled and unveiled Muslim women did not differ significantly regarding body appreciation, it cannot be the veiling per se that led to the higher levels of body appreciation in veiled Muslim women compared to Christian and atheist women. Therefore, future studies should focus on determining which factors might buffer the negative effects of being inactive on the body image of veiled Muslim women.

Regarding the correlations between engagement in sports and body appreciation as well as self-esteem, the findings were mixed. In line with previous research [13], positive correlations between engagement in sports, body appreciation, and self-esteem were found for veiled Muslim women and Christian women, but not for unveiled Muslim women and atheist women. A possible explanation for this divergent finding might lie in the measure of self-esteem that was used in the present study, as such a global measure might be influenced by other factors besides the level of physical exercise [71]. For instance, in a study comparing the self-esteem of normal-weight participants, who were divided into sedentary, non-athletes, and athletes, significant group differences only emerged for the appearance-related aspect of self-esteem, and not for the aspects of performance (e.g., evaluating one’s achievements) or social (e.g., being confident regarding one’s public image; [72]). Moreover, research on sports and self-esteem has shown that physical self-esteem, i.e., physical competence, mediated the link between engaging in sports and global self-esteem [73]. Therefore, future studies should replicate the associations between engagement in sports, body appreciation, and self-esteem found in correlation analyses, by using measures that focus on physical self-esteem [71]. Nevertheless, significant correlations were found between body appreciation and self-esteem across all groups. Thus, the more body appreciation a woman reported, the greater was her self-esteem, which is in line with previous results [11, 14].

Consistent with previous findings, the four groups in the current study showed comparable levels of drive for thinness [74]. In general, most women want to have a thin body and factors such as ethnicity or cultural background become less important when considering the ideal body size for women [24].

In contrast, and in line with expectation, veiled and unveiled Muslim women reported lower levels of drive for leanness than did Christian women and atheist women. This corresponds to the finding that Muslim women engaged less in sports than did Christian women and atheist women. Possibly, striving for a lean body might be less important for Muslim women compared to Christian women and atheist women, which may explain their lower sporting activity. Furthermore, Muslim women might see lean bodies as less attractive than do Christian women and atheist women. In this context, it is of interest to consider the beauty ideal of Muslim women. On the one hand, veiled and unveiled Muslim women reported similar levels of drive for leanness, and on the other hand, Christian women and atheist women reported similar levels. Accordingly, one might assume that cultural factors could be an underlying reason for the different levels of drive for leanness. Thus, in contrast to the assumed global thin ideal [24], the findings of the present study might not support a global lean ideal. In accordance with research findings that some Muslim people dislike the engagement of Muslim women in sports [32, 39], it is possible that Muslim women do not strive for a lean body, as their environment would dismiss this.

Regarding muscularity, the four groups did not differ significantly in drive for muscularity. Thus, Christian women and atheist women did not strive more for a muscular body than did Muslim women. As previous research found that men showed higher levels of drive for muscularity compared to women [20], it may be the case that having a muscular body is not especially important for most females. However, given the differences (albeit non-significant) in drive for muscularity between Muslim women and non-Muslim women, it may be that the drive for muscularity questionnaire did not sufficiently depict the differences between Muslim and non-Muslim women regarding their drive for a muscular body. In this context, previous researchers have argued that the drive for muscularity questionnaire needs more items which address the body parts that are more important for women, e.g., stomach or thighs [54]. Moreover, as Kertechian and Swami [69] found that veiled Muslim women showed a lower internalization of muscular ideals than did unveiled Muslim women, it is possible that the levels of internalization of muscular ideals might differ between Muslim women, Christian women, and atheist women. Therefore, future studies could repeat the comparisons between Muslim women, Christian women, and atheist women by using the *Internalization: Muscular/Athletic* subscale of the Sociocultural Attitudes Towards Appearance Questionnaire 4.

### Strengths and limitations

A strength of the present study lies in the direct comparisons between veiled and unveiled Muslim women as well as non-Muslim women, i.e., Christian women and atheist women,. Moreover, the current study was the first to investigate drive for muscularity in Muslim women. Further strengths of the study were its large number of participants and the quantitative methodology employed, which is in contrast to the existing qualitative studies with only a small number of Muslim participants. Nevertheless, the study has some limitations, for instance the lack of assessment of extent of religiosity or level of acculturation. Based on the study’s focus on engagement in sports and drive for leanness and muscularity, the assessment of further aspects of religiosity was not seen as essential. From the present findings, it is not possible to draw conclusions about the link between extent of religiosity and engaging in sports or striving for a lean and muscular body, but this did not constitute an aim of the study. As associations between engagement in sports and level of acculturation were found in previous studies (e.g., [75]), future studies might assess participants’ level of acculturation. Another limitation lies in the differing sample sizes in the four subgroups, which may have affected the results insofar as existing differences between the groups did not reach significance. However, given that every subgroup consisted of at least fifty participants, the conditions for the respective calculations were more than met. Despite this, it would be desirable for future studies to include similar sample sizes in the respective subgroups. Furthermore, as we used Facebook and mailing lists to recruit participants, it might be that the women in the sample predominantly have a high affinity for technology. Lastly, it is possible that some women did not only think of female bodies when answering items such as “I think the best looking bodies are well-toned.” but instead had male bodies in mind, which may have distorted their results. Therefore, in future studies, the respective instructions should be more explicit and participants might be asked whether they thought of male bodies when answering the questionnaires.

### Implications of the present study

The current study aimed to draw comparisons between veiled and unveiled Muslim women, Christian women, and atheist women regarding engagement in sports, body appreciation, drive for leanness, and drive for muscularity. The findings give rise to the following theoretical implications: As veiled Muslim women, but not unveiled Muslim women, reported higher body appreciation than did Christian women and atheist women, future studies should investigate the factors that may have enhanced the body image of veiled Muslim women. As a negative body image is a risk factor for the development of an eating disorder [12], it is important to ascertain possible protective factors against a negative body image. Furthermore, future studies might examine why veiled and unveiled Muslim women reported lower levels of drive for leanness but not of drive for muscularity compared to Christian and atheist women, e.g., by interviewing Muslim women about their individual beauty ideals. Moreover, as veiled and unveiled Muslim women, Christian women, and atheist women did not differ significantly in drive for muscularity, future studies should address the question of whether a global muscularity ideal for the female body size—similar to that which is assumed for thinness [24]—might exist.

Finally, the following practical implications arise from the current results: Firstly, reasons for the lower levels of engagement in sports among Muslim women living in Germany should be determined in order to diminish barriers to their engagement in sports. For instance, sporting facilities which are suitable for veiled and unveiled Muslim women could be established in order to enable a larger number of Muslim women to participate in sporting activities [4]. Secondly, practitioners like teachers, physicians, or psychologists could educate Muslim women and especially veiled Muslim women about the positive health effects of engaging in sports. In this context, it might be conducive to mention that according to Islam, all Muslims should be fit and healthy, and these goals can be supported by doing sports [4, 31, 67].

## Conclusion

The current study was the first to investigate drive for leanness and drive for muscularity in Muslim women. Moreover, it compared engagement in sports between veiled and unveiled Muslim women, Christian women, and atheist women. Results showed that veiled and unveiled Muslim women had lower levels of drive for leanness but not of drive for muscularity than did Christian women and atheist women. Furthermore, compared to Christian and atheist women, a higher percentage of veiled and unveiled Muslim women did not engage in sports. Therefore, reasons for the higher levels of non-engagement in sports among Muslim women should be determined in order to diminish barriers to their engagement in sports. In addition, future studies might investigate why veiled and unveiled Muslim women reported lower levels of drive for leanness but not of drive for muscularity and could examine whether a global muscularity ideal for the female body size might exist.

## Supplementary Information

**Additional file 1**. Engagements in sports.

## Data Availability

The questionnaire and datasets used and/or analysed during the current study are available from the corresponding author on reasonable request.

## Abbreviations

e.g.: For example
i.e.: Id est (Latin)
etc.: Etcetera
M: Mean score
SD: Standard deviation
