# Supplementary material for: Are there associations between religious affiliation and drive for muscularity? A cross-sectional survey of young Muslim women, Christian women and atheist women from Germany
Source: BMC Womens Health. 2020 Dec 9;20:271. doi: 10.1186/s12905-020-01138-8 (PMC7727221; doi:10.1186/s12905-020-01138-8)
Supplement: Supplementary file 1 — Additional file 1. Engagements in sports. [file 12905_2020_1138_MOESM1_ESM.docx]

**Additional File 1**

**Engagement in sports**

Types of sports (it was possible to select more than one option or to add a non-listed type)

□ Fitness (e.g., Pilates, Yoga, Zumba)

□ Endurance sport (e.g., jogging, swimming, spinning)

□ Weight training (e.g., body weight exercises, weightlifting)

□ Team sport (e.g., soccer, handball, rugby, volleyball)

□ No sports

Other: ____________________

Amount of exercise per week

□ < 1 times

□ 1-3 times

□ 4-6 times

□ > 6 times

Average duration of exercise

□ < 30 minutes

□ 31-60 minutes

□ 61-120 minutes

□ > 120 minutes
